# Supplementary material for: Mutations of Rad6 E2 ubiquitin-conjugating enzymes at alanine-126 in helix-3 affect ubiquitination activity and decrease enzyme stability
Source: J Biol Chem. 2022 Sep 23;298(11):102524. doi: 10.1016/j.jbc.2022.102524 (PMC9630792; doi:10.1016/j.jbc.2022.102524)

Supplementary Figure 1

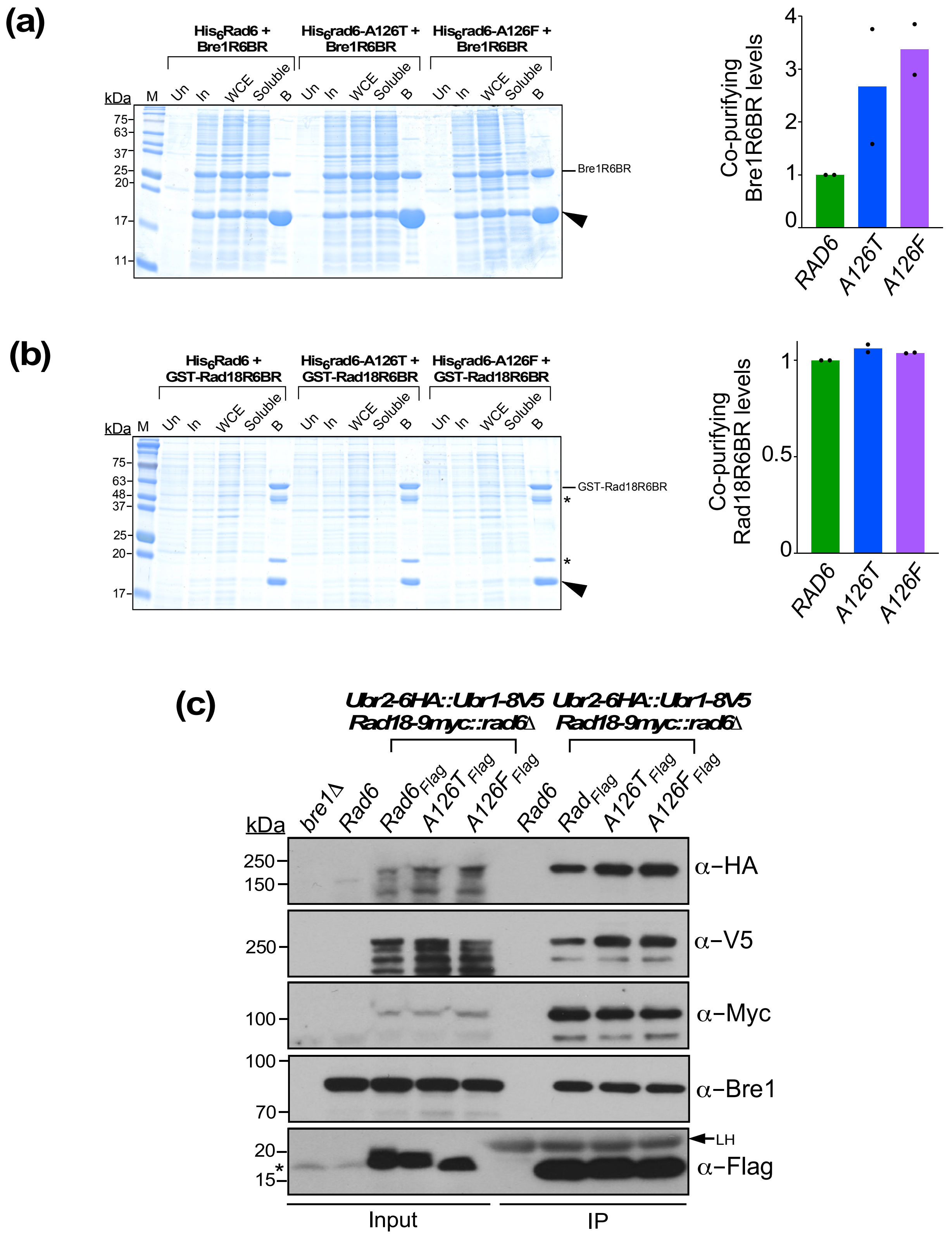

Supplementary Figure 2

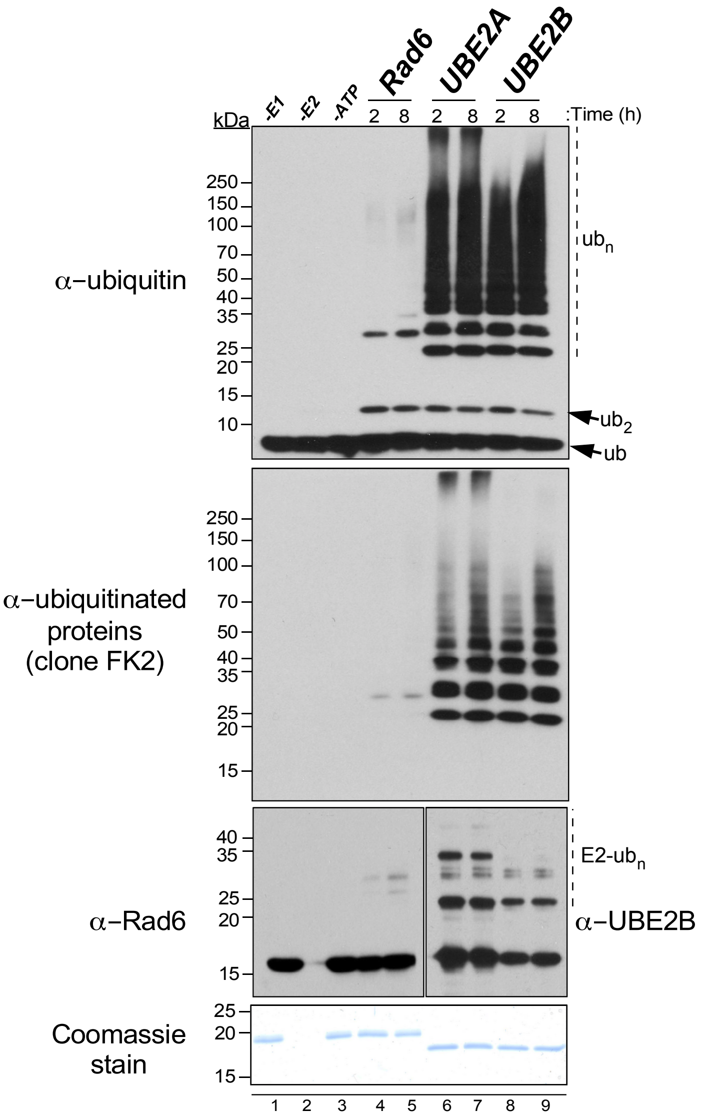

Supplementary Figure 3

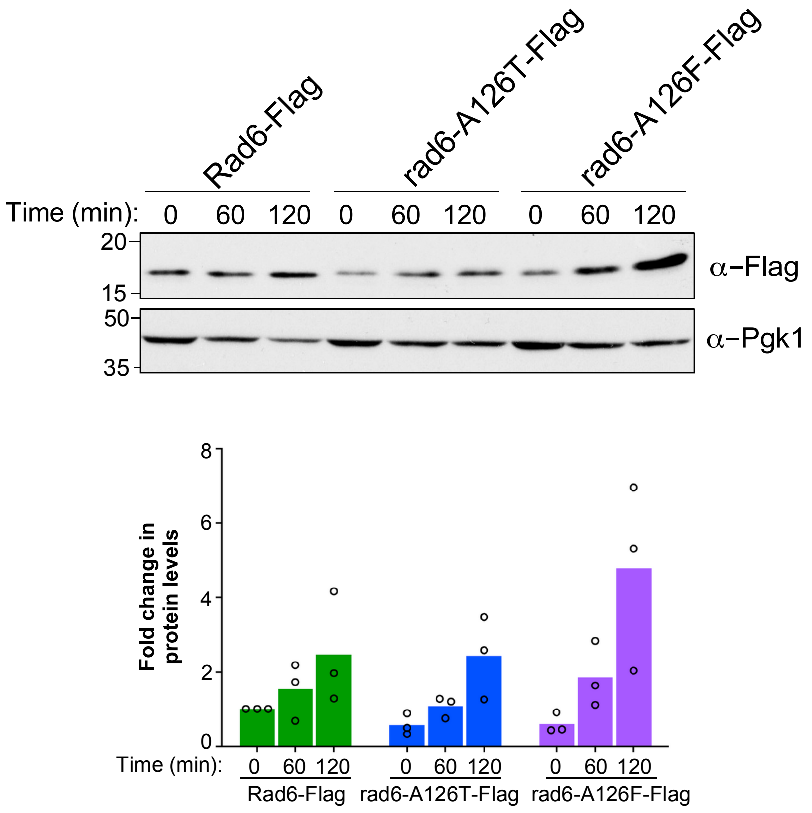

Supplementary Figure 4

(a)

```
sp|P06104|UBC2_Scer      MSTPARRRLMRDFKRMKEDAPPGVSASPLPDNVMVWNAMIIGPADTPYEDGTFRLLLEFD
sp|P49459|UBE2A_Hsap     MSTPARRRLMRDFKRLQEDPPAGVSGAPSENNIMVWNAVIFGPEGTPFEDGTFKLTIEFT
sp|P63146|UBE2B_Hsap     MSTPARRRLMRDFKRLQEDPPVGVSGAPSENNIMQWNAVIFGPEGTPFEDGTFKLVIEFS
                         ***************::** * ***.:*   :*:* ***:*:** .**:*****:* :**

sp|P06104|UBC2_Scer      EEYPNKPPHVKFLSEMFHPNVYANGEICLDILQNRWTPTYDVASILTSIQSLFNDPNPAS
sp|P49459|UBE2A_Hsap     EEYPNKPPTVRFVSKMFHPNVYADGSICLDILQNRWSPTYDVSSILTSIQSLLDEPNPNS
sp|P63146|UBE2B_Hsap     EEYPNKPPTVRFLSKMFHPNVYADGSICLDILQNRWSPTYDVSSILTSIQSLLDEPNPNS
                         ******** *:*:*:********:*.**********:*****:*********:::*** *

sp|P06104|UBC2_Scer      PANVEAATLFKDHKSQYVKRVKETVEKSWEDDMDDMDDDDDDDDDDDDDEAD
sp|P49459|UBE2A_Hsap     PANSQAAQLYQENKREYEKRVSAIVEQSWRDC--------------------
sp|P63146|UBE2B_Hsap     PANSQAAQLYQENKREYEKRVSAIVEQSWNDS--------------------
                         *** :** *::::* :* ***.  **:**.*
```

α−helix-3

(b)

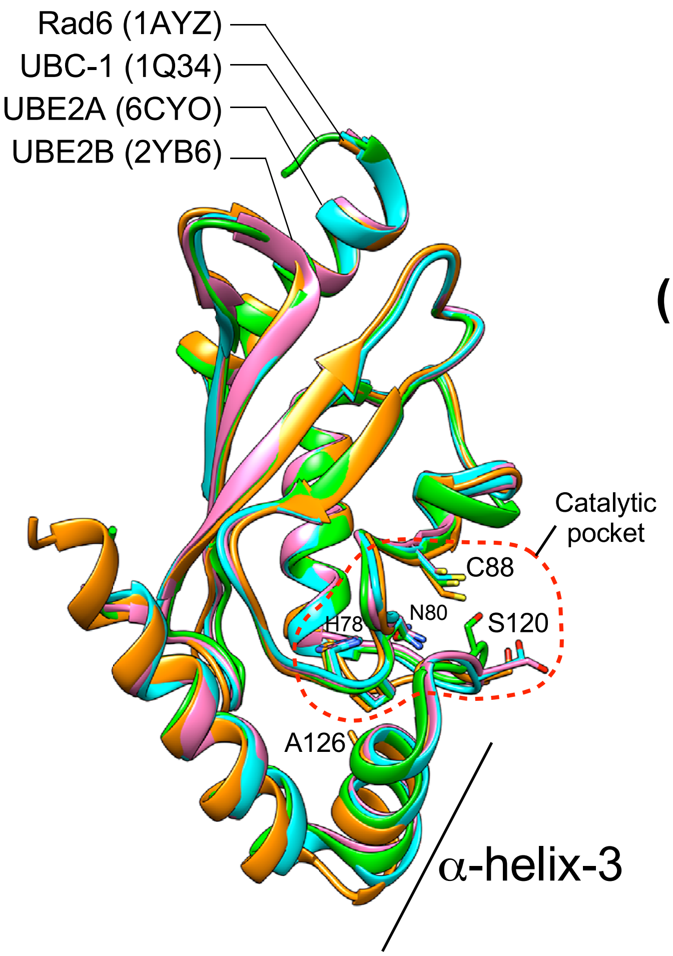

(c)

```
           124 125 126 127 128 129 130 131 132
Rad6:  . . V   E   A   A   T   L   F   K   D  . .
UBE2A: . . S   Q   A   A   Q   L   Y   Q   E  . .
UBE2B: . . S   Q   A   A   Q   L   Y   Q   E  . .
               :   *   *       *   :   :   :
```

α−helix-3

Supplementary Figure 5

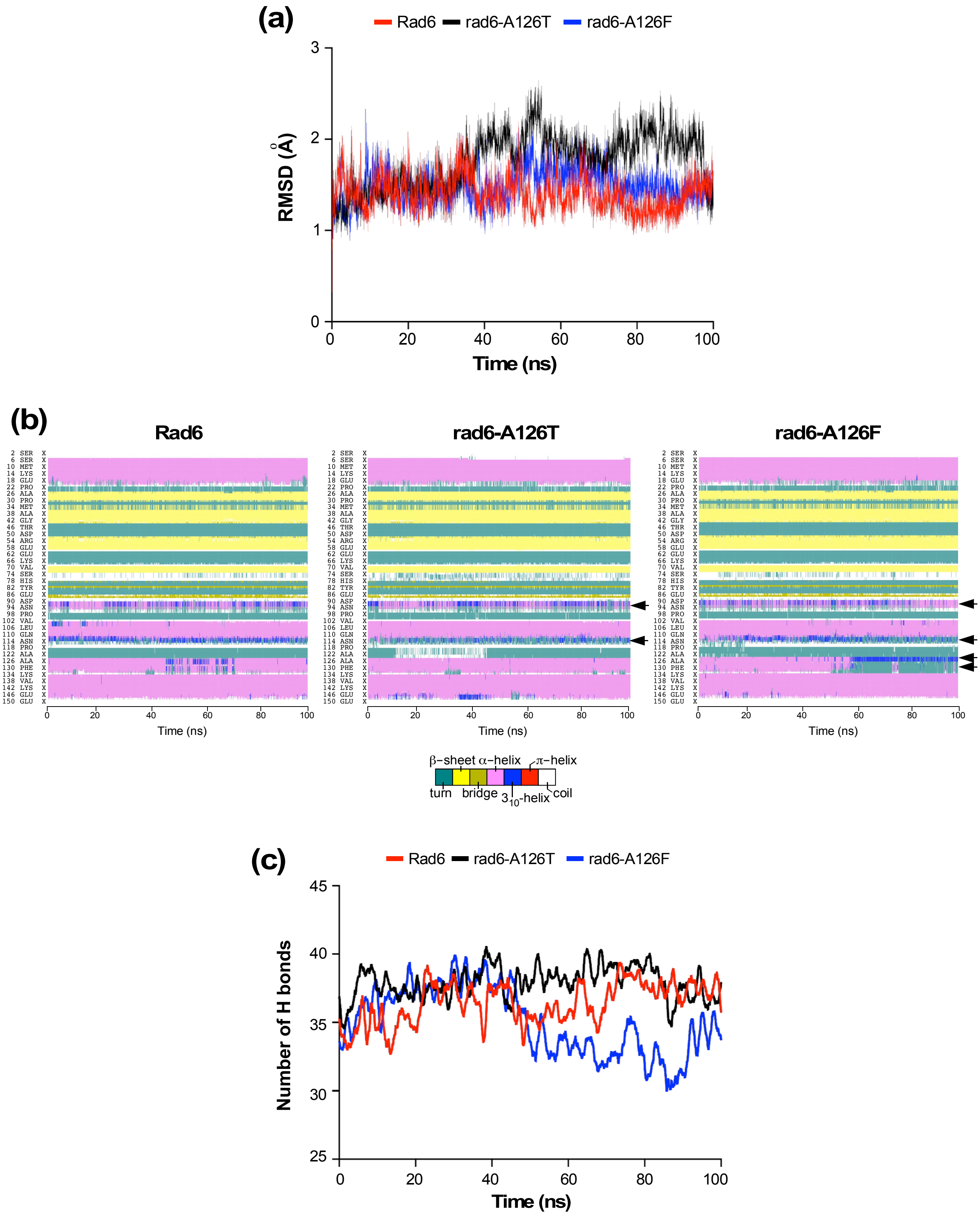

Supplementary Figure 6

(a)

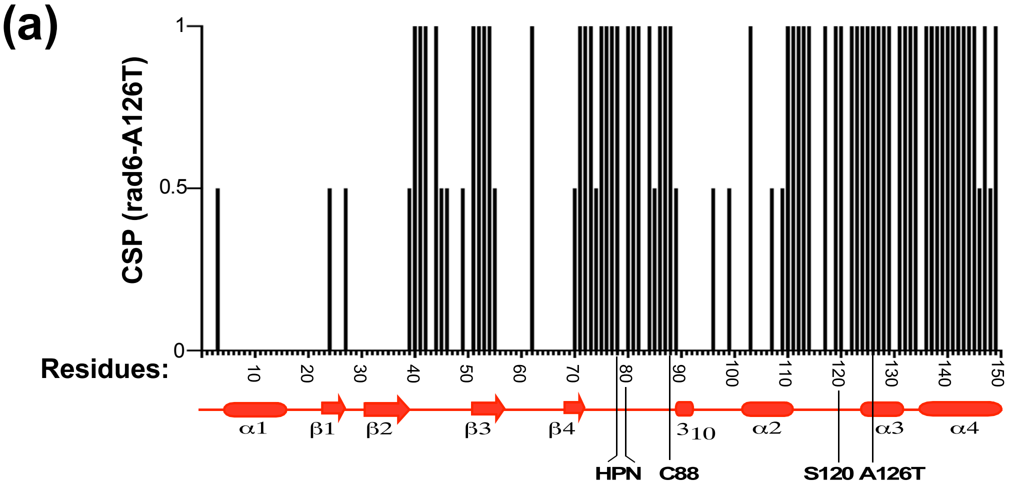

(b)

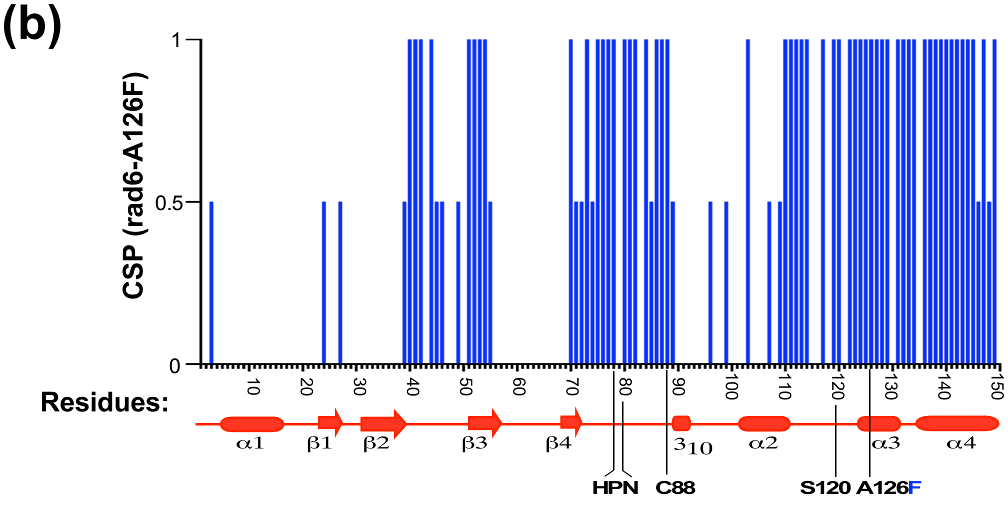

Supplementary Figure 7

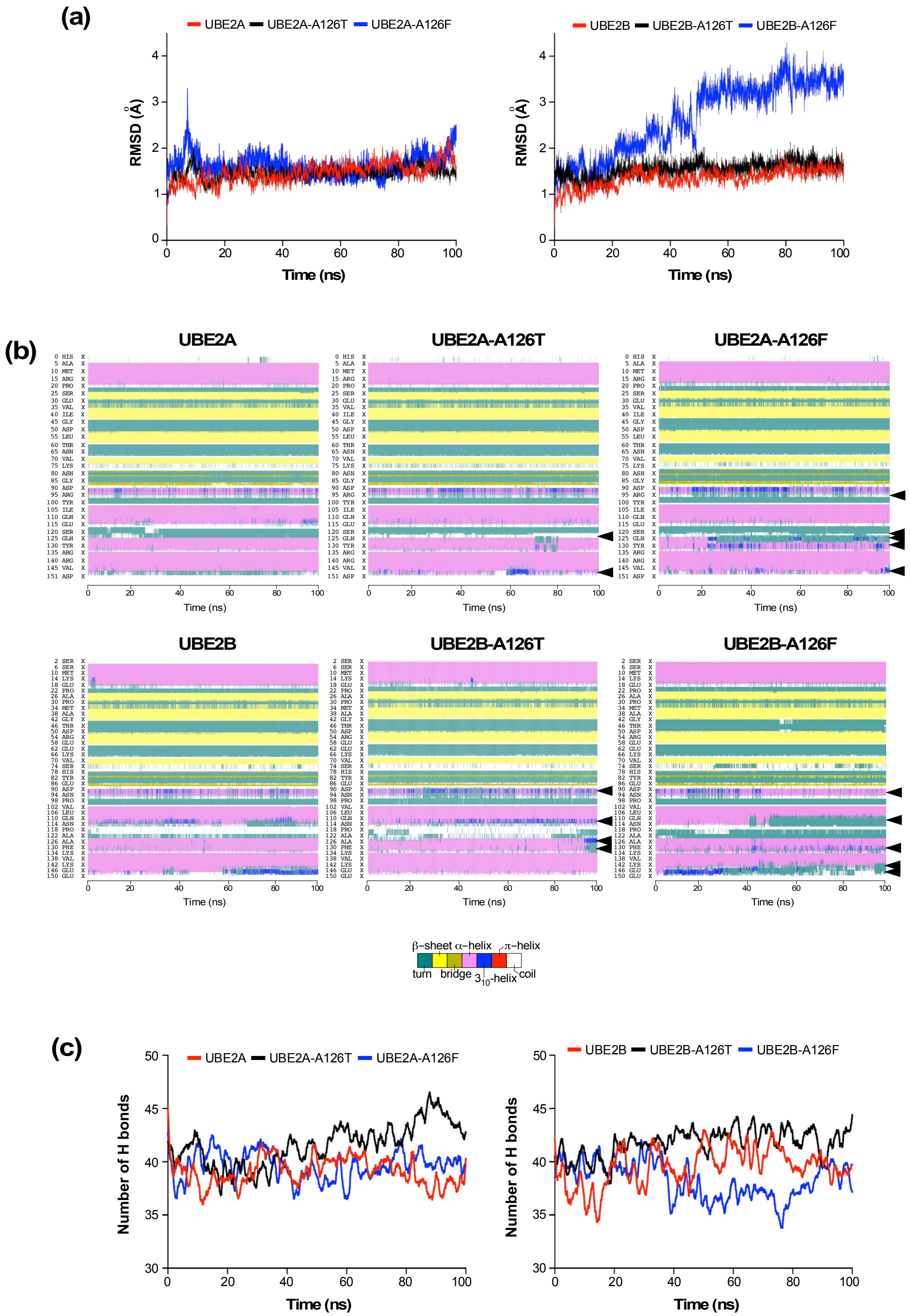

Supplementary Figure 8

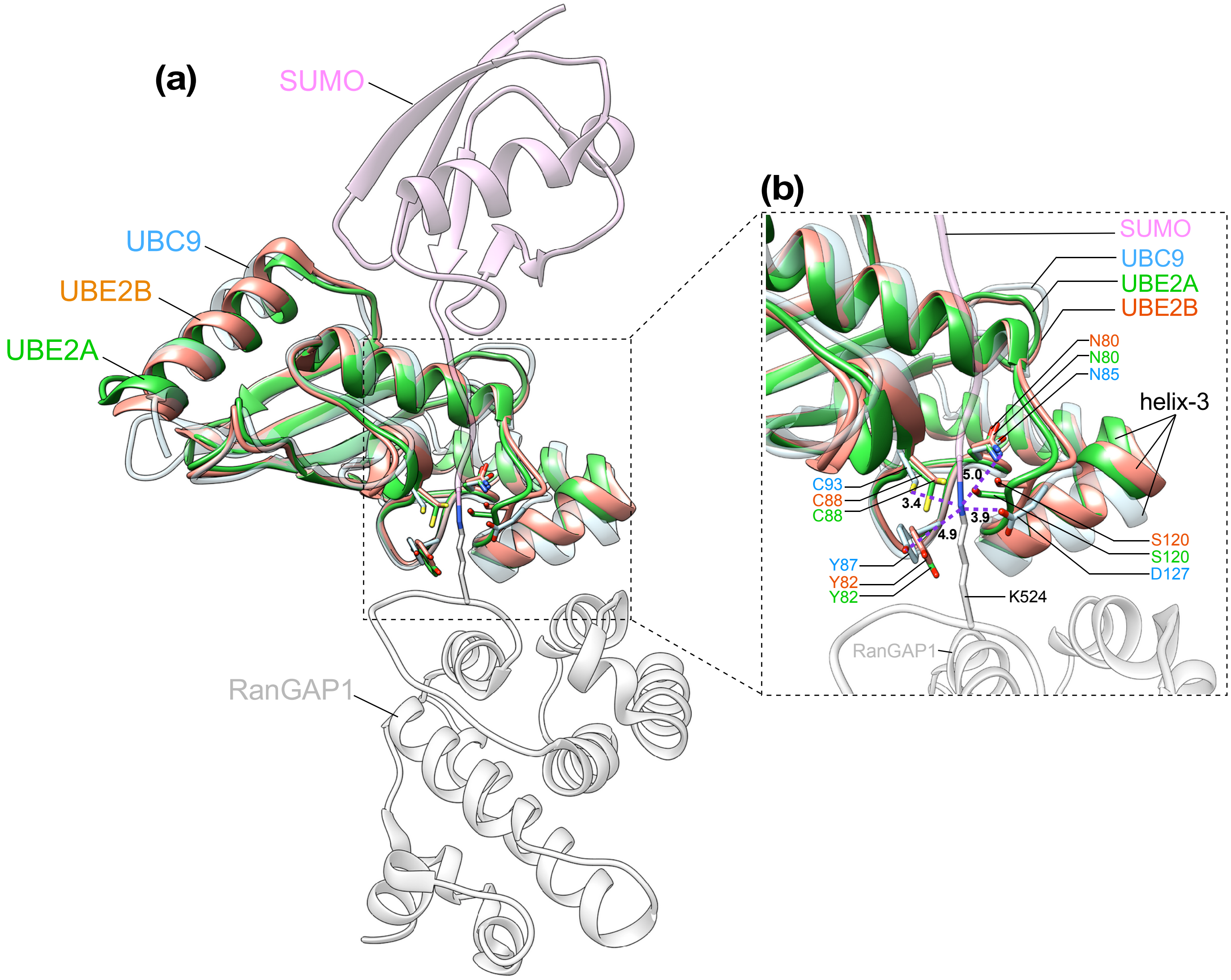

Supplement: Supplemental Figure S1 — Interactions with partner E3 ubiquitin-ligases are not impaired by Rad6 A126 mutations.A and B, left, SDS-PAGE of lysates from uninduced (UIn) and IPTG-induced (In) bacterial cells, whole-cell lysates (WCL), and soluble and bound (B) fractions from cells coexpressing His6-Rad6 or His6-rad6-A126T or His6-rad6-A126F and i) Bre1R6BR or ii) GST-tagged Rad18R6BR. R6BR, Rad6 binding region. The arrowheads indicate His6Rad6 or mutant. Asterisks indicate truncated product. Right, histograms of average levels of copurifying Bre1R6BR or GST-Rad18R6BR normalized to the levels of partner wild-type or mutant Rad6 (two independent experiments). C, co-immunoprecipitation (IP) experiment. Immunoblots of lysates from yeast strains expressing Flag-tagged Rad6 or indicated mutants with 6HA-tagged Ubr2, 8V5-tagged Ubr1, or and 9Myc-tagged Rad18. The input was 5%of the lysate. Lysate from bre1Δ strain or strain expressing proteins without epitope tags served as controls for the anti-Bre1 and epitope-tag specific antibodies, respectively. The asterisk indicates cross-reacting protein. The arrow marked LH indicates immunoglobulin light chain. Supplemental Figure S2. Time-course in vitro ubiquitin chain formation assay was performed using recombinant yeast Rad6 or human homologs UBE2A or UBE2B. Yeast or human Uba1 and ubiquitin were used with yeast Rad6 or its human homologs, respectively. Control reactions were performed without yeast Uba1 (-E1), without Rad6 (-E2), or without ATP. Blots were probed with antibodies recognizing ubiquitin, mono- or poly- ubiquitinated proteins (clone FK2), Rad6 or UBE2B. Note that the anti-UBE2B antibody recognizes UBE2A. Ub, ubiquitin; Ub2, diubiquitin; Ubn, ubiquitin chains or polyubiquitinated Rad6 or UBE2A or UBE2B; E2-Ubn, ubiquitinated Rad6 or its human homologs. Coomassie-stained gel shows the amount of Rad6, UBE2A or UBE2B used in each reaction. Supplemental Figure S3. Yeast strains with Flag-tagged wild-type Rad6 or an A126 mutant were treated with [file mmc1.pdf]
