# Supplementary material for: Mutations of Rad6 E2 ubiquitin-conjugating enzymes at alanine-126 in helix-3 affect ubiquitination activity and decrease enzyme stability
Source: J Biol Chem. 2022 Sep 23;298(11):102524. doi: 10.1016/j.jbc.2022.102524 (PMC9630792; doi:10.1016/j.jbc.2022.102524)
Supplement: Supplemental Tables S1 and S2 [file mmc2.docx]

**Table S1.** Folding free energy (∆∆G) and stability predictions using Dynamut 2.0

| **Protein** | **Mutation** | **∆∆Gstability (kcal/mol)** | **Stability change** |
| --- | --- | --- | --- |
| Rad6 | A126T | –1.22 | Destabilizing |
|  | A126F | –0.62 | Destabilizing |
| UBE2A | A126T | –1.49 | Destabilizing |
|  | A126F | –0.80 | Destabilizing |
| UBE2B | A126T | –1.83 | Destabilizing |
|  | A126F | –0.73 | Destabilizing |

Table S2. Yeast strains list

**Strains Genotype**

YMH171^1^ *Mata* *ura3-52 leu2-3,112 his3 trp1∆*

DHY214^2^ *Mata* *his3∆* *leu2∆* *ura3∆* *lys2∆*

DHY217^2^ *Mata his3∆* *leu2∆ura3∆* *arg4∆*

GAC202a^3^ *Mata his3-11 leu2-3,11 ura3-52 lys2-801 trp1-1 pdr5∆:: KanMX6 pre3-∆2::HIS3 pup1∆::leu2-HIS3 [pRS317-pup1-T30A [YCplac22-pre3-T20A] gal−*

YZS375 *Mat*a *ura3-52 leu2-3,112 his3 trp1∆* *rad6∆::KanMX6*

YZS377 *Mata ura3-52 leu2-3,112 his3 trp1∆ TEL-VII-URA3 rad6∆::KanMX6*

YZS413 *Mat*a *ura3-52 leu2-3,112 his3 trp1∆* *bre1∆::KanMX6*

W4622-14B^4^ *Mata ade2-1 can1-100 his3-11,15 leu2-3,112 trp1-1 ura3-1 RAD5 + YFP-8ala-SML1::ADE2*

YMC162 *Mata ade2-1 can1-100 his3-11,15 leu2-3,112 trp1-1 ura3-1 RAD5 + YFP-8ala-SML1::ADE2 rad6∆::KanMX6*

YMC309 *Mata his3∆* *leu2∆* *ura3∆* *arg4∆* *ubp8∆::KanMX6 ubp10∆::NATMX rad6∆::URA3*

YMC315 *Mata ura3-52 leu2-3,112 his3 trp1∆ UBR2-6HA::HIS3 RAD18-9MYC::NATMX 8V5-UBR1::LEU2 rad6∆::URA3*

YMC336 *Mata his3∆* *leu2∆* *ura3∆* *arg4∆* *ubp10∆::NATMX rad6∆::URA3*

1. Zhang, Y., Sun, Z. W., Iratni, R., Erdjument-Bromage, H., Tempst, P., Hampsey, M., and Reinberg, D. (1998) Mol. Cell 1, 1021-1031.
2. Kindly provided by Late Joe Horecka
3. Collins G.A., Gomez T.A., Deshaies R.J., Tansey W.P. (2010) Yeast. 27(11): 965-974.
4. Andreson, B.L., Gupta A., Georgieva, B.P., Rothstein, R. (2010) Nucleic Acids Res. 38(19): 6490-6501.
